# Chest wall stabilization and rib fixation using a nitinol screwless system in selected patients after blunt trauma: long-term results in a single-centre experience

**DOI:** 10.1093/icvts/ivab278

**Published:** 2021-10-22

**Authors:** Aljaz Hojski, Arben Xhambazi, Mark Nikolaj Wiese, Dragan Subotic, Helga Bachmann, Didier Lardinois

**Affiliations:** Department of Thoracic Surgery, University Hospital Basel, Basel, Switzerland

**Keywords:** Rib fixation, Nitinol device, Pain control, Health-related quality of life

## Abstract

**OBJECTIVES:**

First experiences with rib fixation using nitinol, in terms of reliability and morbidity, influence on pain control and quality of life (QOL), in a large series of selected patients after blunt chest trauma.

**METHODS:**

Data of all patients who had undergone rib fixation by the use of nitinol were retrospectively analysed in terms of indications, morbidity and in-hospital mortality. Pain status and health-related QOL were assessed preoperatively, when possible, at discharge and at 1, 3, 6 and 12 months post-surgery using visual analogous scale and short form 12 questionnaires.

**RESULTS:**

From September 2017 to April 2019, 70 patients underwent rib fixation using the nitinol device, of which 47 (67%) had dislocated, painful fractures, 6 (8.5%) had flail chest injuries, 6 (8.5%) were emergencies with haemodynamical instability and 11 (16%) had pseudoarthrosis. Morbidity was 21% without wound infection; in-hospital mortality was 3%. Fracture of the material occurred in 6% of the patients during the first year, but removal of the material was not required. Analysis of the pain score showed a statistically significant decrease in pain for both the whole collective and the group with a series of dislocated and painful fractured ribs (*P* < 0.001, Tukey contrast on the linear mixed-effects models). Assessment of health-related QOL revealed a significant improvement in the physical score for the mid- and long-term analysis.

**CONCLUSIONS:**

Our results suggest that rib fixation using the nitinol device is reliable, associated with an acceptable morbidity, while significantly decreasing pain and improving health-related QOL.

**Figure:**
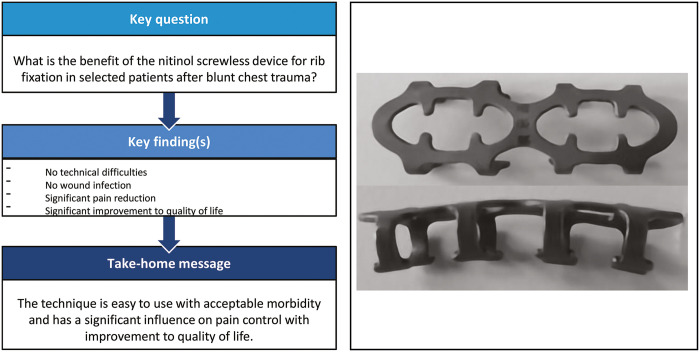

## INTRODUCTION

Treatment of rib fractures associated with blunt chest trauma is still commonly conservative [1]. However, conservative management can be associated with complications in the acute post-traumatic phase, due to its duration or to its inefficacity [2]. Complications consist of infections and prolonged respiratory failure, leading to prolonged hospitalization and a high mortality rate, depending on other injuries, age and comorbidities. In addition, several reports have shown that conservatively treated rib fractures can be accompanied by the impairment of daily activities and quality of life due to persistent pain or chest wall deformity, fracture non-union and chest tightness [3, 4]. Bhatnagar *et al.* [5] observed an average loss of 70 days of work during recovery after conservative therapy of flail chest. Landercasper *et al.* [6] described a long-term disability in up to 60% of patients. However, the level of evidence in the published data is relatively low due to the heterogeneity of the techniques used and the comparatively small number of patients included in most of the studies. Prospective randomized trials, several systematic reviews, meta-analyses and monocentric studies have shown that early stabilization of flail chest injuries can improve the incidence of pneumonia, need for tracheostomy, duration of intubation, length of stay in intensive care unit and in hospital in comparison with conservative therapy [7–15].

**Figure 1:**
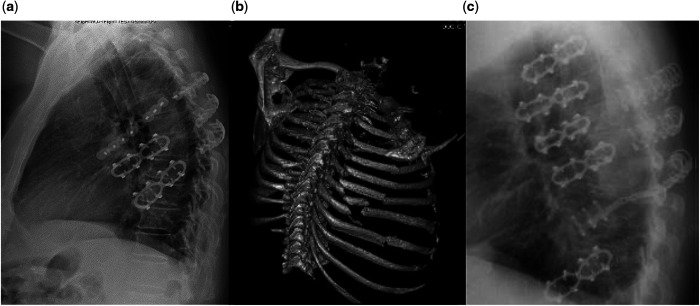
Illustration of the combination of different systems to obtain the optimal results in complex situations. Chest X-radiation with plates and screws (Synthes^®^, Biomet^®^) and with nitinol clips (Europrisme^®^) (**A**); 3-dimensional reconstruction examination with a series of dislocated plurifragmentary rib fractures (**B**); and chest X-radiation after stabilization using nitinol clips (Europrisme) and titanium clips (MedXpert^®^) (**C**).

In recent years, the interest in stabilization of the chest wall in flail chest patients or in rib fixation has grown considerably. This is evident in the development of new surgical fixation devices and in the creation of several professional training courses on chest wall surgery with the purpose of making the indications and the techniques more familiar to the surgical community. Concerning the technique used for rib fixation, several systems are available (Fig. 1). Most of the modern devices are made of titanium, which allows optimal adaptation to the anatomy of the ribs and ensures good protection against infection. Systems with plates as well as screwless devices are available. A device made from nitinol (alloy of titanium and nickel) has recently been launched (central picture). This system is based on thermic properties (shape-memory effect) as the material is malleable at temperatures between 0 and 5°C. After application to the fracture, simple contact with a warm dressing allows its return to the initial hard position, thus providing stability (Video 1). The material exhibits high elasticity, some 10–30 times that of ordinary metal, and as such has the potential of offering the optimal method for surgical stabilization. As the number of patients surgically treated with this material in our department is one of the highest worldwide, we would like to publish our first experiences from a consecutive series of patients with blunt chest trauma.

## MATERIALS AND METHODS

### Ethical statement

This study was approved by the Ethikkommission Nordwest- und Zentralschweiz on 17 December 2018 with the Registration No. EKNZ 2018-02225. No specific individual informed consent was required.

In September 2017, we introduced a new screwless system, the NiTi Fixing Plates^®^ (Europrisme^®^, Luxembourg) to perform rib fixation and chest wall stabilization. This system has been available for commercial use in Switzerland from August 2016. All the patients included in this study had suffered a blunt chest trauma and would have had to undergo surgery irrespective of the method used. This means that the nitinol device had absolutely no influence on the indication for surgery nor on the pre- and postoperative clinical management of the patients. About 400 patients with rib fractures are referred to our centre each year.

Medical data were collected from all patients in whom rib fixation or chest wall stabilization by using nitinol clips alone or in combination with other devices had been performed between 1 September 2017 and the end of April 2019. One of the main study objectives was to analyse the clinical outcome of the procedures performed using this new device. For this purpose, we have retrospectively harvested general information about patient history, clinical data, duration of surgery, length of hospital stay, morbidity and mortality. Clinical and radiological examinations were performed at predetermined times (Table 1). Information about pain status and subjective health-related quality of life (HRQOL) was collected before surgery, if possible, at hospital discharge and at 1, 3, 6 and 12 months after surgery (Table 1). A visual analogous scale (VAS) was used to assess the pain score related to the thoracic injury. For some patients with flail chest injuries or who had undergone emergency surgery, no pain score could be obtained preoperatively due to intubation on admission. HRQOL was assessed using the short form 12 health survey version 2 questionnaire, which is a standardized and validated shortened version of the short form 36 form [16]. The 12 questions are assigned to 8 scales measuring physical and mental health. The possible range of physical component summary (PCS) and mental component summary (MCS) score is 0–100, a higher PCS or MCS score indicating a better HRQOL [16]. An analysis of the costs was not within the scope of this study. Nevertheless, we can state that, in our centre, the overall costs of the nitinol device are comparable to the other systems (both with screws and screwless).

**Table 1:** Standard assessment of patients undergoing rib fixation or chest wall stabilization after blunt trauma

	Preoperative	At discharge	At 1-, 3-, 6- and 12-month post-surgery
Clinical examination	+	+	+
Wound/scar control	‒	+	+
Chest X-ray	‒	+	+
Chest CT scan	+	‒	‒
Pain score	(+)	+	+
HRQOL	(+)	+	+

(+): when possible, according to the indication for surgery.

CT: computed tomography; HRQOL: health-related quality of life; X-ray: X-radiation.

Statistical analysis was performed for the specified observation time points (preoperative and 3, 6 and 12 months post-surgery) for all patients and for the group of dislocated, painful fractures. For the 3 other indications, statistical analysis was not performed because of the low number of patients in these groups. Cronbach’s alpha was calculated to estimate the internal consistency of the short form 12 questionnaire, with values between 0.84 and 0.91 for all 4 time points showing very stable results. As verified by quantile comparison plots, pain score, PCS and MCS were analysed on the original scale, fulfilling the conditions of an approximately Gaussian distribution of the residuals. Changes over time were evaluated separately for pain score, PCS and MCS by 3 analogous linear mixed-effects models with the preoperative time point as reference. *P*-values were calculated by applying the Tukey contrast to the mixed-effects models. The regression models were adjusted for age and gender. The estimates of the regression models showed the difference of mean values together with the corresponding 95% confidence intervals and *P*-values. A *P*-value of <0.05 was considered significant. Analysis was performed with the statistical program R version 3.5.1 [17].

Depending on the surgical technique, the approach was always as minimally invasive as possible. In flail chest patients, a long incision was avoided to minimize the trauma to the contused soft tissue. Two small incisions were preferred, enabling the fixation of up to four ribs. After mobilization of the muscles, the fractures were identified. Care was taken to avoid injuring the neurovascular intercostal bundle, and the periosteum was not dissected. The rib width was measured, and the appropriate nitinol clip was chosen from the device range, i.e. ∼20% smaller than the measured width. The device was then cooled down for ∼1–3 min in a refrigerated sterile physiological solution. Each pair of gripping arms was successively opened with a dedicated instrument. After placing the nitinol clip onto the fracture, the device was warmed using a dressing soaked in a physiological solution of 45°C. The clip adapted automatically to the rib, returning to its initial shape after closure of the arms (Video 1).

## RESULTS

Between September 2017 and the end of April 2019, 109 patients [78 (72%) men and 31 (28%) women] with a mean age of 64.7 years (19–96) underwent chest wall stabilization or rib fixation using NiTi Fixing Plates following a blunt chest trauma. Thirty-nine patients were excluded from the analysis: 12 patients had not provided general informed consent for research, 26 were surgically treated with a combination of nitinol clips and other systems (MatrixRib^®^, Synthes, Switzerland; Stratos^®^, MedXpert, Germany) and 1 patient underwent rib fixation after an iatrogenic rib fracture during surgery. The analysis was performed on those 70 patients who provided general informed consent for research and were surgically treated with the nitinol system only. The indications for surgery were a dislocated and painful series of fractured ribs despite adequate pain therapy (47 patients), flail chest in the absence of extended lung contusion or cerebral injuries (6 patients), emergency treatment of haemothorax with haemodynamical instability (6 patients) and pseudoarthrosis (11 patients). Twenty-two patients included in the study had associated injuries, consisting of 6 patients with additional abdominal and pelvic trauma, 8 patients with head and/or spine injuries and 15 patients with trauma of the extremities.

**Figure 2:**
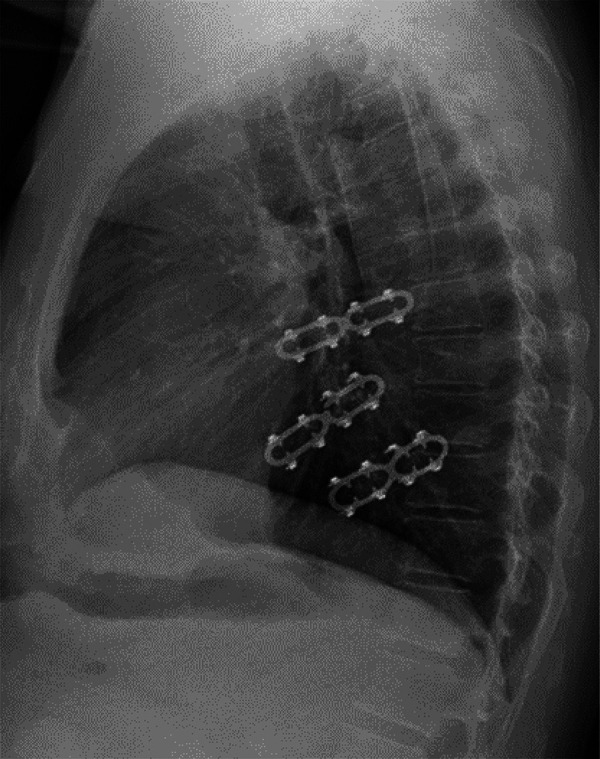
Chest X-radiation 3-month post-surgery showing fracture of 1 clip without dislocation of the rib fractures.

Data regarding duration of surgery, time interval between trauma and operation and hospitalization time are shown in Table 2. No patient in this series required a bilateral fixation of ribs. The operation was performed on the right side in 50% of patients. Injury of the diaphragm was observed in 6 (9%) patients, requiring emergency surgery for haemothorax. Bleeding could be controlled thoracoscopically in all patients. The mean number of fractured ribs was 5.9 (1–15). In total, 241 nitinol clips were used in 70 patients with a mean number of 3.4 (1–10) clips per patient. In-hospital complications were observed in 15 (21%) patients, consisting of 5 cases of pneumonia, 1 of pneumothorax, 4 of post-surgery delirium, 2 of seroma, 2 of haemothorax requiring reoperation, 1 gastritis requiring gastroscopy, 1 fracture of the hip following a fall from the bed and 1 wound haematoma. No wound infection occurred. Three patients with pneumonia were treated successfully with aggressive bronchial toilet and antibiotics. The 2 other pneumonia patients died during hospitalization due to multi-organ failure, resulting in an in-hospitalization mortality rate of 2/70 (3%). Post-surgery delirium was seen only in very old patients. Patients with seroma required no reoperation. Wound haematoma developed in 1 patient, and wound revision with vacuum assisted closure therapy was performed 8 days post-surgery. No microorganism could be identified, and wound healing was uneventful after the administration of broad-spectrum antibiotics for 14 days. Three-month follow-up showed an uneventful scar. Two patients developed a haemothorax post-surgery. One patient underwent revision 1 day after rib fixation for dislocated and painful rib fractures, the second patient underwent thoracoscopy 1 week after fixation for pseudoarthrosis. Recovery was uneventful in both patients. At 3-month follow-up, fractures of the material occurred in 2/70 (3%) patients and a total of 2 clips (2/241, 1%) were broken. At 6 months, fractures of the material were observed overall in 4/70 (6%) patients and a total of 4 clips (4/241, 2%) were broken (Fig. 2). At 1 year post-surgery, no additional fractures of the material were observed. All fractures occurred on the back of the clip, leaving the pairs of arms intact. In all patients, fracture of the device occurred without evident new trauma. There was no dislocation of the broken material, and no removal was required.

**Table 2:** Duration of surgery, time interval between trauma and operation, hospitalization time according to the indication for surgery [mean value (standard deviation) number of patients]

	Operation duration (min)	Time interval between trauma and operation (days)	Hospitalization time (days)
All patients	107 (40) 70	82 (201) 70	14 (9) 70
Dislocated, painful fractures	109 (37) 47	14 (17) 47	15 (9) 47
Flail chest	112 (51) 6	35 (58) 6	21 (11) 6
Emergency	120 (29) 6	13 (12) 6	8 (4) 6
Pseudoarthrosis	91 (53) 11	437 (334) 11	6 (2) 11

Regarding pain analysis, a preoperative assessment of the VAS score could be obtained in 67 patients. The VAS score showed a progressive decrease in pain during evolution in all subgroups of patients (Table 3). In both the ‘all patients’ and the ‘dislocated and painful series of fractured ribs’ groups, a statistically significant decrease in the pain score was observed in the comparison of preoperative and post-surgery VAS scores (*P* < 0.001, Tukey contrast on the linear mixed-effects models, Table 6). Analysis of HRQOL assessed by the short form 12 questionnaire is summarized in Tables 4 and 5. The analyses of both PCS and MCS showed a worsening of the HRQOL immediately after surgery, but a progressive improvement was observed between the first check-up after surgery and up to 12 months for the whole study group. In the group of dislocated, painful fractures, an improvement in PCS was also confirmed at 6 and 12 months compared to the preoperative score (Table 6).

**Table 3:** Descriptive analysis of pain levels as per VAS score in the 70 patients undergoing rib fixation only with the nitinol system [mean (SD) patients]

	All patients	Dislocated, painful fractures	Flail chest	Emergency	Pseudoarthrosis
Preoperative	7.5 (2.4) 67	8.3 (1.5) 47	7.0 (4.1) 5	6.4 (2.6) 5	5.0 (2.8) 10
At discharge	3.6 (1.5) 66	3.6 (1.6) 47	4.6 (1.1) 5	2.5 (0.6) 4	3.7 (1.5) 10
1-Month post-surgery	2.8 (1.5) 63	2.7 (1.6) 45	2.8 (2.2) 5	2.7 (2.1) 3	3.2 (0.9) 10
3-Month post-surgery	2.0 (1.8) 61	2.0 (1.7) 43	2.8 (3.8) 4	0.8 (0.5) 4	2.3 (0.9) 10
6-Month post-surgery	1.8 (1.9) 52	1.4 (1.5) 37	3.0 (3.6) 3	1.3 (2.3) 3	2.8 (2.7) 9
12-Month post-surgery	1.6 (1.9) 50	1.5 (1.9) 35	1.5 (1.9) 4	2.5 (3.5) 2	2.1 (2.0) 9

Mean values represent scores ranging from 0 to 10. A higher score corresponds to greater pain levels.

SD: standard deviation; VAS: visual analogous scale.

**Table 4:** Descriptive analysis of the SF-12 questionnaire physical score (PCS) in the 70 patients undergoing rib fixation only with the nitinol system [mean (SD) patients]

	All patients	Dislocated, painful fractures	Flail chest	Emergency	Pseudoarthrosis
Preoperative	39.5 (11.1) 56	40.5 (11.4) 39	32.5 (10.1) 4	41.8 (12.9) 3	38.0 (10.2) 10
At discharge	36.1 (7.4) 53	36.0 (7.7) 37	29.0 (4.6) 4	37.4 (4.1) 3	39.0 (6.6) 9
1-Month post-surgery	39.3 (7.0) 58	39.0 (7.2) 42	42.4 (4.5) 4	38.1 (5.1) 2	39.5 (7.6) 10
3-Month post-surgery	41.2 (8.0) 52	40.8 (8.6) 37	42.4 (2.1) 3	38.7 (5.4) 3	43.0 (7.7) 9
6-Month post-surgery	45.0 (8.9) 48	46.2 (8.8) 34	43.2 (13.2) 3	48.1 (9.1) 2	40.4 (7.7) 9
12-Month post-surgery	46.7 (8.3) 43	47.3 (9.0) 28	44.4 (7.2) 4	44.9 (7.4) ) 2	46.2 (7.6) 9

PCS: physical component summary; SD: standard deviation; SF-12: short form 12.

**Table 5:** Descriptive analysis of the SF-12 questionnaire mental score (MCS) in the 70 patients undergoing rib fixation only with the nitinol system [mean (SD) patients]

	All patients	Dislocated, painful fractures	Flail chest	Emergency	Pseudoarthrosis
Preoperative	46.5 (11.5) 56	47.2 (12.9) 39	42.3 (10.8) 4	45.5 (0.5) 3	45.8 (7.0) 10
At discharge	41.4 (7.2) 53	41.4 (6.7) 37	37.2 (6.5) 4	35.7 (9.5) 3	45.5 (7.8) 9
1-Month post-surgery	43.6 (9.3) 58	44.3 (9.1) 42	45.8 (3.9) 4	35.3 (15.2) 2	41.8 (10.7) 10
3-Month post-surgery	47.4 (10.3) 52	48.7 (9.3) 37	31.6 (14.5) 3	45.5 (8.7) 3	48.3 (10.8) 9
6-Month post-surgery	49.8 (10.4) 48	52.8 (8.8) 34	41.3 (15.6) 3	47.7 (2.5) 2	42.1 (11.2) 9
12-Month post-surgery	52.3 (9.0) 43	53.3 (8.1) 28	50.7 (17.5) 4	50.3 (5.2) 2	50.6 (8.6) 9

MCS: mental component summary; SD: standard deviation; SF-12: short form 12.

**Table 6:** Multiple comparisons by the Tukey contrast of the linear mixed-effects models predicting either the VAS score, SF-12 questionnaire physical score (PCS) or mental score (MCS) for all patients and the group dislocated, painful fractures

	All patients			Dislocated, painful fractures		
	VAS	PCS	MCS	VAS	PCS	MCS
3 months versus preoperative	*P* < 0.001	*P* = 0.82	*P* = 0.86	*P* < 0.001	*P* = 1.0	*P* = 0.89
6 months versus preoperative	*P* < 0.001	*P* = 0.003	*P* = 0.057	*P* < 0.001	*P* = 0.033	*P* = 0.028
12 months versus preoperative	*P* < 0.001	*P* < 0.001	*P* = 0.003	*P* < 0.001	*P* = 0.018	*P* = 0.065

MCS: mental component summary; PCS: physical component summary; SF-12: short form 12; VAS: visual analogous scale.

## DISCUSSION

Fixation of rib fractures is still a matter of debate. Some prospective monocentric studies have described a positive long-term outcome after chest wall stabilization in patients with flail chest with a 100% return-to-work rate at 2 months with a 95–100% working capacity and no restriction in lung function test at 6 months [18–21]. Majercik *et al.* [22] observed no residual pain after fixation as early as 5.4 weeks post-surgery with a mean satisfaction score of 9.2 on a visual scale.

Our first experience with the nitinol device is positive. The technique is remarkably simple, quick and reproducible. This could be an advantage in comparison to plates and screws. In our patients, the mean surgery duration can be considered comparatively long. This is mainly due to the use of thoracoscopy in most patients or to combined procedures with the trauma surgeons in the case of additional injuries. We observed no infection, which correlates with the low rate found by Thiels *et al.* [23] (0–10%). In the patients with seroma, no removal of the material was required, which could guarantee the further stability of the chest wall and avoid progressive dislocation of the ribs. We observed 4/70 (6%) patients with fractures of material after 1 year corresponding to a fracture rate of 2% of the used clips. Ruptures of titanium-made materials have already been described, sometimes leading to reoperation because of pain or instability of the chest wall [24]. With the nitinol alloy, such fractures were not expected, since the alloy is highly elastic and should not suffer fatigue. An explanation of the observed fractures could be the impossibility to modify the shape of the clip because of its shape-memory properties. It means that the device is not perfectly shaped for all the anatomical aspect of the ribs. In the 4 patients with fractured clips, the rib fractures were not ideally located anterolaterally but rather in the lateral and posterolateral aspect of the ribs. For the comparatively rare indication of fractures localized in this portion of the ribs, we would therefore recommend the use of more bendable systems. Another potential disadvantage of the nitinol system is the availability of only one length of plate (50 mm) with 4 pairs of arms. This might be too short to correctly cover and fix complicated fractures with multiple fragments or oblique fractures. In such a situation, it is potentially possible to apply 2 clips close to each other or to use another system. These 2 observations explain why in some patients we have used a combination of several devices, which also shows the advantage of having several systems available in order to offer patients the optimal solution depending on the localization of the fractures and of the quality of the bone (Fig. 1).

The mean duration of hospitalization was 15 days for patients with dislocated and painful rib fractures. This can be considered long, but most of these patients had first been offered conservative therapy, with surgery only proposed several days after admission. Another explanation of the prolonged hospitalization time is that many patients in Switzerland usually go to a rehabilitation clinic after discharge from hospital. They are obliged to go there directly without a transitory stay at home, thereby prolonging their hospitalization. In recent years, the indication for rib fixation has been extended to selected patients without flail chest.

Two recently published smaller studies suggest that patients benefit from rib fixation in terms of pain control and functional results [25, 26]. In our study, the subgroup of patients with dislocated, painful fractures was by far the largest, corresponding to 67% of the indications for surgery. Our initial policy had been to consider first an adequate pain therapy, consisting of different possible combinations of oral drugs, patient-controlled anaesthesia pump or peridural analgesia. After 4–5 days, rib fixation was discussed for patients with persisting pain and dislocated ribs. Analysis of the pain score showed a positive effect of the procedure even in the early post-surgery phase, with a clear reduction in all indication groups. An important finding was not only the early pain reduction in the group of patients with dislocated series of fractures, confirming the first published data, but also the continuation of the surgery’s positive effect in long-term observation, probably justifying a more liberal approach to rib fixation in selected patients of this group. Based on our growing positive experience, we have changed our policy and now advise surgery earlier for selected patients to reduce hospitalization time, especially for young active patients who wish to be fit again as soon as possible and also for selected older patients who are living on their own, independently and who are still active with a good quality of life.

Our study also showed the positive effect of surgery with the nitinol system on HRQOL. It is the first report of a mid- and long-term benefit on patients’ post-surgery HRQOL. In the descriptive analysis, both PCS and MCS were improved for up to 12 months post-surgery in all patients as well as in each subgroup of indications. Interestingly, at the time of discharge, both scores were lower than preoperatively, probably due to several factors such as the trauma itself, the operation, associated injuries and the hospitalization. But at the first follow-up 1 month after surgery, both scores had already improved. Statistical analysis confirmed the analysis of the pain score in all patients and in the main subgroup, also showing a statistically significant improvement of PCS in comparison to the preoperative score at mid- and long-term follow-up (Table 6). The short-term analysis at 3 months showed no statistical significance, probably because despite significant pain reduction, complete physical and mental recovery required a little bit longer than 3 months in our patient collective.

### Limitations

Our study has several limitations due to its retrospective design without a comparison arm. Although to our knowledge, it is the largest study on rib fixation with the nitinol system to date, the number of patients remains comparatively small. A complete statistical analysis was possible only in the total group and in the subgroup of dislocated, painful rib fractures. HRQOL analyses are difficult to evaluate as they are influenced by an abundance of factors. Not only rib fixation but also associated injuries, other invasive procedures, hospitalization and rehabilitation, as well as personal background, have an impact on HRQOL.

## CONCLUSION

In conclusion, our study shows that chest wall stabilization or rib fixation with the nitinol screwless system is safe and easy to use in different indication groups. The rate of broken material is a little disturbing, but in none of the cases, this complication had a detrimental effect on the well-being of the patients or required reoperation. It indicates room for further improvement to the system, underscoring the advantage of access to a range of fixation systems. Our report demonstrates a clear post-surgery benefit in selected patients in terms of short-, mid- and long-term pain control and in the mid- and long-term improvement to quality of life. The data available in the literature on conservative therapy with a high incidence of long-term disability together with the clear results from our study might point to the need of a prospective study comparing conservative therapy and surgery in selected patients with painful and dislocated rib fractures.

## Notes

Presented at the 27th European Conference on General Thoracic Surgery, Dublin, Ireland, 9–12 June 2019.

## ABBREVIATIONS

HRQOL: Health-related quality of life
MCS: Mental component summary
PCS: Physical component summary
QOL: Quality of life
VAS: Visual analogous scale
